# Improvement in 5-Year Relative Survival in Cancer of the Corpus Uteri From 1993–2000 to 2001–2006 in Japan

**DOI:** 10.2188/jea.JE20170008

**Published:** 2018-02-05

**Authors:** Shusaku Inoue, Satoyo Hosono, Hidemi Ito, Isao Oze, Yoshikazu Nishino, Masakazu Hattori, Tomohiro Matsuda, Isao Miyashiro, Tomio Nakayama, Mika Mizuno, Keitaro Matsuo, Kiyoko Kato, Hideo Tanaka, Yuri Ito, the J-CANSIS Research Group

**Affiliations:** 1 Division of Molecular and Clinical Epidemiology, Aichi Cancer Center Research Institute, Nagoya, Japan; 2 Division of Epidemiology and Prevention, Aichi Cancer Center Research Institute, Nagoya, Japan; 3 Department of Epidemiology and Public Health, Kanazawa Medical University, Ishikawa, Japan; 4 Department of Cancer Therapy Center, Fukui Prefectural Hospital, Fukui, Japan; 5 Cancer Information Services and Surveillance Division, Center for Cancer Control and Information Services, National Cancer Center, Tokyo, Japan; 6 Cancer Control Center, Osaka International Cancer Institute, Osaka, Japan; 7 Department of Gynecology, Aichi Cancer Center Hospital, Nagoya, Japan; 8 Department of Gynecology and Obstetrics, Graduate School of Medical Sciences, Kyushu University, Fukuoka, Japan; 9 Department of Epidemiology, Nagoya University Graduate School of Medicine, Nagoya, Japan; 10 Osaka Prefectural Health Center, Osaka, Japan

**Keywords:** cancer registry, cancer of the corpus uteri, population-based study, prognosis, relative survival

## Abstract

**Background:**

Medical circumstances in Japanese patients with cancer of the corpus uteri have greatly changed since the late 1990s, including the introduction of concomitant therapy with taxane and platinum. We evaluated changes in survival rates for this cancer following these advances by analyzing data from population-based cancer registries in Japan.

**Methods:**

Data were available for 8562 cases of cancer of the corpus uteri from six prefectural cancer registries. We defined the two periods of 1993–2000 (1st period) and 2001–2006 (2nd period). Relative survival (RS) in each period was calculated to assess changes using an excess mortality model, with adjustment for age group (15–54, 55–69, and 70–99 years), extent of disease (localized, regional, and distant), and histological subtype.

**Results:**

Overall 5-year RS improved from 77.7% in the 1st period to 80.2% in the 2nd period, with an excess hazard ratio (EHR) of 0.785 (95% confidence interval [CI], 0.705–0.873). Five-year RS significantly improved in the group aged 55–69 years, in all groups by extent of disease, and in the endometrioid adenocarcinoma group. In particular, 5-year RS significantly improved in patients with endometrioid adenocarcinoma, from 84.5% to 89.7%, with an EHR of 0.698 (95% CI, 0.560–0.870).

**Conclusion:**

Overall 5-year RS for cancer of the corpus uteri in Japan improved from the 1990s to early 2000s. These improvements might have been aided by the comprehensive medical development of management for this cancer, including the spread of concomitant therapy with taxane and platinum as a standard adjuvant chemotherapy in the early 2000s.

## INTRODUCTION

In developed countries, treatment for cancer of the corpus uteri changed from the late 1990s to early 2000s with the introduction of concomitant therapy with taxane and platinum. The Surveillance, Epidemiology, and End Results Program (SEER) in the United States reported a 5-year relative survival (RS) for this cancer of 83.3% from 2005 to 2011,^1^ while a European Cancer Registry-based study reported a rate of 76.2% from 2000 to 2007.^2^ In Japan, 5-year RS using subjects registered with a Japanese population-based registry was 79.8% between 2003 and 2005.^3^ Cancer of the corpus uteri is generally recognized as having a favorable prognosis because most patients are diagnosed at an early stage.^4^ Nevertheless, approximately 320,000 people were diagnosed with this cancer worldwide in 2012 and 76,000 died.^5^ Respective numbers for Japan in 2011 were about 14,700 newly diagnosed cases^6^ and 2,000 deaths.^7^ Given this high incidence, investigating the degree of improvement in survival due to the spread of effective therapeutic approaches and diagnostic modalities as observed in population-based studies is an important public health issue. Although population-based studies of survival trends for this cancer have been reported in Japan and other developed countries, no study has detailed changes following improvements in treatment, including the introduction of concomitant therapy with taxane and platinum.

The most important treatment modality for cancer of the corpus uteri is surgery.^8^ After initial surgery, the need for adjuvant therapy is determined based on a risk assessment of recurrence.^9^ More than 40% to 50% of patients diagnosed with this cancer in Japan required adjuvant therapy during the period of the present study.^10^^,^^11^ The main adjuvant therapy regimen in the 1990s was anthracyclines and platinum-based anti-cancer drugs. Concomitant therapy with taxane and platinum for this cancer was first reported in the late 1990s.^12^ Despite the lack of convincing evidence from a phase III trial, the regimen became widespread in the early 2000s and was used as first-line adjuvant chemotherapy in more than half of Japanese patients in 2004.^13^ However, evidence to recommend its use as adjuvant therapy is still insufficient. Although a small number of randomized control trials (RCTs) have investigated the efficacy of the regimen, their results were controversial.^14^ Due to the characteristically favorable prognosis of cancer of the corpus uteri, it is difficult to confirm the difference between postoperative adjuvant therapies in RCT settings. Instead, determining the impact of these dramatic changes in chemotherapy regimen over the past two decades on survival might be better achieved using a population-based study with a large study population.

In the same period, several advances in other treatment approaches or development of other medical circumstances also occurred in Japan. For example, the change of postoperative adjuvant therapy occurred, and many supporting therapies for chemotherapy were developed. Further, peri-operative care, including anesthesiology, markedly advanced, and some imaging modalities, such as transvaginal sonography, computed tomography, and magnetic resonance imaging, spread widely.

Here, we conducted a population-based survival analysis of cancer of the corpus uteri in Japan following these developments in management, with stratification by age group, extent of disease, and histological subtype at year of diagnosis.

## MATERIAL AND METHODS

### Population

Our research project, the Japanese Cancer Survival Information for Society (J-CANSIS), was established in 2013 to analyze recent trends in cancer survival and report long-term survival based on population-based cancer registry data in Japan. As part of this project, we analyzed RS for cancer of the corpus uteri (International Classification of Disease, 10th revision [ICD-10] code: C54) among 23 cancer sites^15^ using anonymized data of 8,562 cases diagnosed between 1993 and 2006 obtained from the population-based cancer registries of six prefectures (Yamagata, Miyagi, Fukui, Niigata, Osaka, and Nagasaki). The population covered in our study represented 13.4% of the total Japanese population in 2005. These prefectural cancer registries have long been used to estimate national statistics for cancer survival in Japan, and their cancer record data quality is considered to be high, as evidenced by death certificate notification and death certificate only ratios for all cancers during 1993–2006 of 21.9% and 13.1%, respectively. All subjects were followed by the six cancer registries for at least 5 years after diagnosis using linkage to a death certificate database in the prefecture to confirm the vital status of patients. The registries of Yamagata, Fukui, and Osaka Prefectures for the whole period, as well as Nagasaki Prefecture for part of this period, additionally confirmed the vital status of patients who were anticipated to be alive 5 years after diagnosis using linkage to their residential databases. This method can complement data on patients who move outside the prefecture where they were registered. Loss to follow-up for patients with cancer of the corpus uteri was 2.31%. Survival was calculated based on the number of months from cancer diagnosis to the date of death. Our study was approved by the institutional review board of Osaka Medical Center for Cancer and Cardiovascular Diseases.

### Extent of disease

For extent of disease, patients were classified into the three stages of localized, regional, and distant disease groups. Patients classified into the localized group showed the presence of cancer within the uterine corpus only, with no invasion into adjacent tissues or organs (International Federation of Gynecology and Obstetrics [FIGO] 1988 stage I). Patients classified into the distant group had distant metastases (FIGO 1988 stage IVb). The other patients were classified into the regional group (FIGO 1988 stage II-IVa).

### Histology

For histology, disease codes were based on the International Classification of Diseases for Oncology, 3rd ed. (ICD-O-3), and patients were divided into six histological subgroups (adenocarcinoma, other specified carcinoma, unspecified carcinoma, sarcoma, other specified malignant neoplasm, or unspecified malignant neoplasm) according to modified Cancer Incidence in Five Continents Vol. X published by International Agency for Research on Cancer. We then divided adenocarcinoma into endometrioid adenocarcinoma; other specified adenocarcinoma; and adenocarcinoma, not otherwise specified (NOS), and then separately calculated the RS of carcinosarcoma from those of other specified malignant neoplasms. Accordingly, the classified histological subtypes were endometrioid adenocarcinoma (8380 and 8382); other specified adenocarcinoma (8141, 8210, 8211, 8260, 8262, 8263, 8310, 8384, 8430, 8441, 8460, 8480, 8482, 8560, 8570, and 8572); adenocarcinoma, NOS (8140); other specified carcinoma (8041, 8050, 8051, 8070, 8072, 8075, 8120, 8240, 8246, 8255, and 8323); unspecified carcinoma (8010 and 8020); sarcoma (8800, 8801, 8811, 8890, 8891, 8895, 8896, and 8900); carcinosarcoma (8980); other specified malignant neoplasm (8720, 8930, 8931, 8933, 8935, 8950, 8951, 9085, 9100, 9101, and 9364); and unspecified malignant neoplasm (8000 and 8001).

### Statistical analysis

Frequency distributions between categorical variables were compared using the chi-square test. We estimated RS by period of diagnosis, age group, extent of disease, and histological subtype. RS is the survival rate adjusted for competing causes of death in members of the general population of the same age (ie, the ratio of the observed and the expected survival estimated by background mortality). We used the complete national population life tables defined by sex, single year of age, and single calendar year to derive the background mortality of cancer patients. To provide sufficient statistical power and assess the impact of the introduction of concomitant therapy with taxane and platinum, we divided the period of cancer diagnosis into two ranges, 1993–2000 (1st period, *n* = 3,981) and 2001–2006 (2nd period, *n* = 4,581), in accordance with the timing of introduction of these regimens in Japan. Using a cohort approach, 1-year and 5-year RS were calculated for each period of diagnosis. We applied the maximum likelihood method of Esteve et al to estimate RS.^16^ To compare the differences in RS between the two periods of diagnosis, we applied the excess mortality model, a multivariate regression approach based on generalized linear models, which adopts the Poisson assumption for the observed number of deaths.^17^ The excess hazard ratio (EHR) derived from these models provides the ratio of the hazard of death calculated from the observed to the expected hazard in a given category (2nd period: 2001–2006) to the hazard in the reference category (1st period: 1993–2000), adjusting for age group, extent of disease, and histological subtype. For age groups, patients were classified into three groups aged 15–54 (premenopausal group), 55–69 (postmenopausal group), and 70–99 (elderly group) years. All data management and analyses were carried out using Stata SE Ver. 13.1 (Stata Corp, College Station, TX, USA).^18^

## RESULTS

Study subjects are characterized in Table 1. Median age of all patients was 58 years (range, 19–99 years), and patients aged 55–69 years were the most prevalent in both periods. With regard to the extent of disease, localized cases were the most prevalent, while for histological subtypes, endometrioid adenocarcinoma was the most prevalent except for adenocarcinoma, NOS. The proportion of patients aged 70 years or older and those with endometrioid adenocarcinoma increased from the 1st to the 2nd period, whereas proportions of the extent of disease did not differ between the two periods.

**Table 1.** Characteristics of study subjects

	Year of diagnosis				*P*-value^a^

	1st period (1993–2000)		2nd period (2001–2006)		
	
	*N*	(%)	*N*	(%)	
**Total (*N* = 8,562)**	3,981	(46.5)	4,581	(53.5)	
**Age**					<0.001
≤54 years	1,528	(38.4)	1,681	(36.7)	
55–69 years	1,816	(45.6)	1,948	(42.5)	
≥70 years	637	(16.0)	952	(20.8)	
**Extent**					0.394
Localized	2,501	(62.8)	2,885	(63.0)	
Regional	782	(19.6)	962	(21.0)	
Distant	307	(7.7)	381	(8.3)	
Unknown or missing	391	(9.8)	353	(7.7)	
**Histology**					<0.001
Adenocarcinoma, NOS	1,978	(49.7)	1,167	(25.5)	
Endometrioid adenocarcinoma	1,112	(27.9)	2,339	(51.1)	
Other specified adenocarcinoma	337	(8.5)	446	(9.7)	
Other specified carcinoma	41	(1.0)	51	(1.1)	
Unspecified carcinoma	78	(2.0)	75	(1.6)	
Carcinosarcoma	71	(1.8)	164	(3.6)	
Sarcoma	118	(3.0)	119	(2.6)	
Other specified malignant neoplasm	97	(2.4)	111	(2.4)	
Unspecified malignant neoplasm	149	(3.7)	109	(2.4)	

NOS, not otherwise specified.

^a^chi-square test.

Overall 1-year and 5-year RS by period is shown in Table 2. One-year RS significantly increased from 90.4% in the 1st period to 91.8% in the 2nd period, with an EHR of 0.732 (95% confidence interval [CI], 0.622–0.861). Five-year RS was 77.7% in the 1st period and 80.2% in the 2nd period, showing a significant improvement (EHR 0.785; 95% CI, 0.705–0.873).

**Table 2.** Relative survival rates and estimated excess hazard ratios for cancer of the corpus uteri, 1st period (1993–2000) and 2nd period (2001–2006)

	1-year RS (% with 95% CI)	1-year EHR (95% CI)	*P*-value	5-year RS (% with 95% CI)	5-year EHR (95% CI)	*P*-value
**Overall**						
1st period (*N* = 3,981)	90.4 (89.4–91.3)	1.000		77.7 (76.2–79.1)	1.000	
2nd period (*N* = 4,581)	91.8 (90.9–92.7)	0.732 (0.622–0.861)	<0.001^b^	80.2 (78.9–81.5)	0.785 (0.705–0.873)	<0.001^b^
**Age group**						
**≤54 years**						
1st period (*N* = 1,528)	94.5 (93.2–95.6)	1.000		86.2 (84.2–87.9)	1.000	
2nd period (*N* = 1,681)	94.3 (93.0–95.3)	0.746 (0.528–1.054)	0.097^c^	86.9 (85.1–88.5)	0.816 (0.655–1.018)	0.071^c^
**55–69 years**						
1st period (*N* = 1,816)	89.5 (87.9–90.9)	1.000		73.6 (71.3–75.7)	1.000	
2nd period (*N* = 1,948)	92.6 (91.3–93.8)	0.642 (0.501–0.823)	<0.001^c^	79.9 (77.8–81.7)	0.703 (0.603–0.821)	<0.001^c^
**≥70 years**						
1st period (*N* = 637)	80.2 (76.6–83.4)	1.000		63.4 (58.6–67.8)	1.000	
2nd period (*N* = 952)	83.8 (81.0–86.2)	0.802 (0.606–1.064)	0.126^c^	62.8 (58.9–66.4)	0.905 (0.735–1.115)	0.348^c^
**Extent of disease**^a^						
**Localized**						
1st period (*N* = 2,501)	98.6 (97.9–99.1)	1.000		92.8 (91.5–94.0)	1.000	
2nd period (*N* = 2,885)	99.0 (98.4–99.3)	0.894 (0.518–1.545)	0.689^d^	95.0 (93.9–96.0)	0.757 (0.589–0.973)	0.029^d^
**Regional**						
1st period (*N* = 782)	85.1 (82.3–87.6)	1.000		56.7 (52.9–60.2)	1.000	
2nd period (*N* = 962)	86.5 (84.0–88.6)	0.877 (0.674–1.142)	0.330^d^	63.8 (60.4–67.0)	0.848 (0.723–0.995)	0.044^d^
**Distant**						
1st period (*N* = 307)	43.5 (37.8–49.0)	1.000		18.2 (14.0–22.9)	1.000	
2nd period (*N* = 381)	57.8 (52.6–62.7)	0.660 (0.528–0.826)	<0.001^d^	22.6 (18.4–27.1)	0.791 (0.662–0.945)	0.010^d^

CI, confidence interval; EHR, excess hazard ratio; RS, relative survival rate.

1st period, Patients diagnosed in 1993–2000; 2nd period, Patients diagnosed in 2001–2006.

^a^Extent of disease was unknown or data were missing for 744 cases.

^b^Multivariate model adjusted for age group, extent of disease and histology.

^c^Multivariate model adjusted for extent of disease and histology.

^d^Multivariate models adjusted for age group and histology.

One-year RS in patients aged 55–69 significantly improved from 89.5% to 92.6% (EHR 0.642; 95% CI, 0.501–0.823), while 5-year RS significantly improved from 73.6% to 79.9% (EHR 0.703; 95% CI, 0.603–0.821). In contrast, the 1-year and 5-year RS of patients aged 70 years or older and 54 years or younger did not differ between the periods.

**Figure 1.**
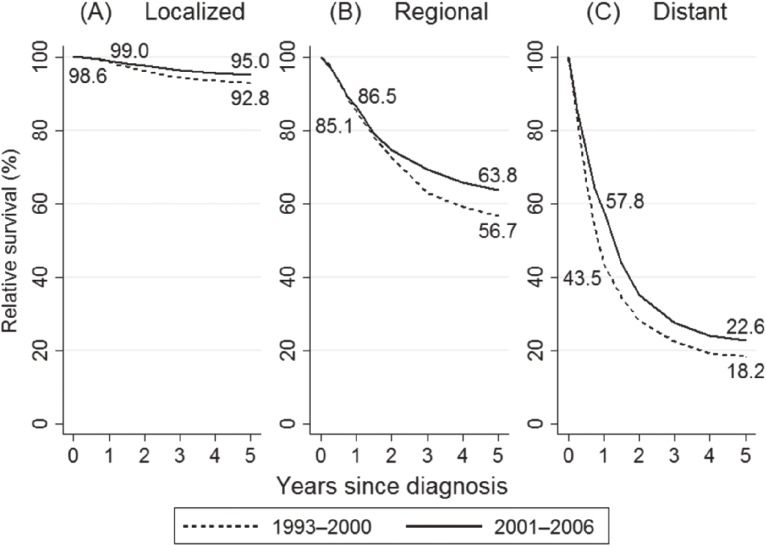
Relative survival of patients with cancer of the corpus uteri by extent of disease in the 1st period (1993**–**2001) and the 2nd period (2001–2006). Dashed line, relative survival curve in the 1st period (1993–2000); solid line, relative survival curve in the 2nd period (2001–2006). (A) RS in the localized group. One-year RS of 1st and 2nd period were 98.6% and 99.0%. Five-year RS of 1st and 2nd period were 92.8% and 95.0%. (B) RS in the regional group. One-year RS of 1st and 2nd period were 85.1% and 86.5%. Five-year RS of 1st and 2nd period were 56.7% and 63.8%. (C) RS in the distant group. One-year RS of 1st and 2nd period were 43.5% and 57.8%. Five-year RS of 1st and 2nd period were 18.2% and 22.6%. RS, relative survival.

Significant improvements in 5-year RS were observed regardless of the extent of disease. In stratified analysis by the extent of disease, 5-year EHR of death in the localized, regional, and distant groups were 0.757 (95% CI, 0.589–0.973), 0.848 (95% CI, 0.723–0.995) and 0.791 (95% CI, 0.662–0.945), respectively. One-year RS among the distant group significantly increased, with an EHR of 0.660 (95% CI, 0.528–0.826), whereas no significant changes were observed among localized and regional groups (Table 2 and Figure 1).

Results by histological subtype are shown in Table 3. Both 1-year and 5-year RS significantly increased in the endometrioid adenocarcinoma group, from 94.0% to 97.0% (EHR 0.598; 95% CI, 0.411–0.868) and from 84.5% to 89.7% (EHR 0.698; 95% CI, 0.560–0.870), respectively. In the other specified adenocarcinoma group, 1-year RS significantly improved from 86.5% to 90.4% (EHR 0.534; 95% CI, 0.331–0.861), although 5-year RS did not improve. One-year RS increased in patients with carcinosarcoma, from 65.9% to 72.1% (EHR 0.558; 95% CI, 0.330–0.944), albeit with no improvement in 5-year RS (eFigure 1).

**Table 3.** Relative survival rates and estimated excess hazard ratios by histology, 1st period (1993–2000) and 2nd period (2001–2006)

	1-year RS (% with 95% CI)	1-year EHR (95% CI)	*P*-value^a^	5-year RS (% with 95% CI)	5-year EHR (95% CI)	*P*-value^a^
**Histology**						
**Endometrioid adenocarcinoma**						
1st period (*N* = 1,112)	94.0 (92.3–95.3)	1.000		84.5 (81.9–86.7)	1.000	
2nd period (*N* = 2,339)	97.0 (96.1–97.7)	0.598 (0.411–0.868)	0.007	89.7 (88.1–91.0)	0.698 (0.560–0.870)	0.001
**Other specified adenocarcinoma**						
1st period (*N* = 337)	86.5 (82.2–89.9)	1.000		69.6 (63.9–74.6)	1.000	
2nd period (*N* = 446)	90.4 (87.0–92.9)	0.534 (0.331–0.861)	0.010	67.5 (62.4–72.0)	0.857 (0.647–1.135)	0.282
**Adenocarcinoma, NOS**						
1st period (*N* = 1,978)	93.8 (92.5–94.9)	1.000		81.5 (79.5–83.4)	1.000	
2nd period (*N* = 1,167)	92.7 (90.9–94.2)	0.912 (0.680–1.222)	0.536	83.0 (80.4–85.4)	0.783 (0.647–0.949)	0.012
**Other specified & unspecified carcinoma**^b^						
1st period (*N* = 119)	77.5 (68.5–84.3)	1.000		67.6 (57.3–75.9)	1.000	
2nd period (*N* = 126)	74.1 (65.2–81.1)	0.658 (0.353–1.224)	0.186	58.4 (48.5–67.0)	0.904 (0.547–1.494)	0.694
**Carcinosarcoma**						
1st period (*N* = 71)	65.9 (53.2–76.0)	1.000		41.7 (29.4–53.6)	1.000	
2nd period (*N* = 164)	72.1 (64.4–78.5)	0.558 (0.330–0.944)	0.030	40.9 (32.8–48.8)	0.706 (0.476–1.046)	0.083
**Sarcoma**						
1st period (*N* = 118)	72.5 (63.3–79.8)	1.000		45.0 (35.5–53.9)	1.000	
2nd period (*N* = 119)	73.2 (64.0–80.3)	0.775 (0.439–1.368)	0.380	37.7 (28.7–46.7)	0.830 (0.556–1.239)	0.362
**Other specified malignant neoplasm**						
1st period (*N* = 97)	81.5 (72.0–88.1)	1.000		64.4 (53.1–73.6)	1.000	
2nd period (*N* = 111)	75.0 (65.5–82.2)	1.030 (0.513–2.066)	0.935	58.4 (48.2–67.3)	1.090 (0.647–1.838)	0.745
**Unspecified malignant neoplasm**						
1st period (*N* = 149)	64.0 (55.3–71.5)	1.000		49.5 (40.6–57.7)	1.000	
2nd period (*N* = 109)	53.1 (42.8–62.4)	0.787 (0.450–1.376)	0.401	37.1 (27.3–46.9)	0.921 (0.565–1.502)	0.742

CI, confidence interval; EHR, excess hazard ratio; RS, relative survival rate.

1st period: Patients diagnosed in 1993–2000, 2nd period: Patients diagnosed in 2001–2006.

^a^Multivariate models adjusted for extent of disease and age group.

^b^Comprising 153 unspecified carcinomas and 92 other specified carcinomas.

## DISCUSSION

In this study using Japanese population-based data, we found that overall 5-year RS in cancer of the corpus uteri significantly improved between patients diagnosed in 2001–2006 and those diagnosed in 1993–2000, with assessment for adjusted EHR. In stratified analysis, a significant improvement was shown in patients aged 55–69 years, in all three groups with regard to extent of disease (localized, regional, distant), and in patients with endometrioid adenocarcinoma. To our knowledge, this is the first study to use population-based data to assess the change in survival in corpus uteri cancer following the changes in management for this cancer in the early 2000s.

Several previous studies have focused on trends in 5-year RS in cancer of the corpus uteri. Using cancer registry data from five northern European countries, Klint et al suggested that survival steadily increased in patients diagnosed during 1964–2003.^19^ They argued that factors behind this steady increase included not only better peri-operative care but also the increased use of hormone replacement therapy, which increased the early detection of cancer of the corpus uteri. A similar analysis of trends in 5-year RS in patients with endometrioid endometrial cancer in the Netherlands by Boll et al showed an increase in RS of 2% during 1989–2003.^20^ The authors commented that the most important reasons for the increase were the increased incidence of FIGO stage I and the increased incidence in endometrioid endometrial cancer of grades 1 and 2. On the other hand, studies based on the SEER data from 1998–2003 and on data from 11 cancer registries in Germany from 2002–2006 found no improvement in 5-year RS.^4^^,^^21^

In Japan, the standardization of treatment substantially progressed from the late 1990s through the early 2000s. First, the chemotherapy regimen shifted from anthracyclines and platinum-based anti-cancer drugs to concomitant therapy with taxane and platinum. Second, adjuvant therapy after initial surgery shifted from radiotherapy to chemotherapy.^13^ In addition, more effective supporting therapies for chemotherapy were developed, and perioperative care, including anesthesiology, advanced markedly. Furthermore, although there was not a particular breakthrough in diagnostic technique in this study period, transvaginal sonography, computed tomography, and magnetic resonance imaging had widely spread in the 2nd period and patients had more opportunities to receive these imaging studies. Thus, more patients were given an accurate diagnosis and an early detection in comparison with the 1st period. In these ways, comprehensive advances in the medical circumstance surrounding patients with this cancer occurred in the study period.

Among them, the changes in chemotherapy regimen and adjuvant therapy might be most relevant. In fact, the annual report of the Japan Society of Obstetrics and Gynecology (JSOG) Committee on Gynecologic Oncology reported a decrease in the use of radiotherapy as postoperative adjuvant therapy from 14.6% to 3.6% over this study period.^10^^,^^11^ In terms of the introduction of novel chemotherapy regimens, concomitant therapy with taxane and platinum for cancer of the corpus uteri was administered for the first time in 1998 in Japan.^12^ Subsequently, a survey of the Japanese Gynecologic Oncology Group in 2004 showed that 65.3% of all patients who underwent adjuvant chemotherapy had already received concomitant therapy with taxane and platinum as the first-line adjuvant chemotherapy.^13^ Generally, indications for chemotherapy were met by some members of the localized group (FIGO 1988 stage Ic) and all members of the regional (FIGO stage II-IVa) and distant groups (FIGO stage IVb), and significant improvements in 5-year RS were accordingly observed in all three groups by extent of disease in our study. In particular, 5-year RS of the regional group improved by a remarkable 7.1% between the two periods. This group was the most likely to be influenced by the shift in adjuvant therapy because all cases in the group require adjuvant therapy, in principle. Furthermore, the contribution of the detection at an earlier stage of disease within the regional group due to frequent use of imaging modalities also cannot be ruled out. A remarkable improvement in 5-year RS was also observed in the endometrioid adenocarcinoma group. Since endometrioid adenocarcinoma was the most common histological subtype, its prognosis substantially affected the overall improvement in 5-year RS. On stratified analysis by age, only the 55–69 years group showed an improvement. The lack of improvement in 5-year RS in the youngest group during the 2nd period may not be surprising, considering that 5-year RS in the 1st period was already favorable. With regard to the elderly group, elderly patients are apt to abandon treatment due to comorbidities or poor general status and might accordingly be less likely to obtain a benefit from the introduction of taxane and platinum concomitant therapy.

This study investigated not only long-term but also short-term RS. One-year RS improved in the age 55–69 years group; the distant group; and the endometrioid adenocarcinoma, other specified adenocarcinoma, and carcinosarcoma groups. In particular, a remarkable improvement in 1-year RS was observed among the distant group, which likely reflects efficacy in the prolongation of survival time rather than in curative function. Similar findings were observed in the carcinosarcoma and other specified adenocarcinoma groups, in which only short-term survival improved. These findings are consistent with reports that the introduction of concomitant therapy with taxane and platinum for highly malignant tumors such as carcinosarcoma and serous adenocarcinoma might be associated with improvements in short-term prognosis,^22^^–^^24^ although the regimen has not been the gold standard for these tumors.

In our study, the survival rates were lower than that of JSOG registry system.^10^^,^^11^ The reason for this survival difference may be mainly explained by the difference in registered subjects. Generally, registered hospitals of the JSOG registry system are core centers providing cutting-edge treatment of cancer. On the other hand, subjects in population-based cancer registries contain all patients in particular geographical regions. These differences in registered subjects may have led the discrepancy between survival rate in population-based cancer registries and that in the JSOG registry system.

There are several strengths and limitations of this study. The study period 1993 to 2006 covered the timing of changes in the treatment of cancer of the corpus uteri in Japan. In addition, our relatively large sample size (8,562 cases) allowed us to investigate the impact of changes with stratification for important prognostic factors, such as age, extent of disease, and histological subtype. However, the large sample size likely lead to the overestimation of EHR, contrary to the difference in RS between the two periods. With regard to limitations, the use of population-based cancer registry data prevented the collection of detailed information on therapy and clinicopathological characteristics. Furthermore, in terms of the extent of disease, we used the classifications from population-based cancer registries due to the absence of information on FIGO staging classification, which may have hampered interpretation of our findings.

In conclusion, we identified a significant improvement in 5-year RS in Japanese patients with cancer of the corpus uteri from 1993–2000 to 2001–2006. Five-year RS was significantly improved in the endometrioid adenocarcinoma group, all disease extent groups, and the group aged 55–69 years. Further, 1-year RS was significantly improved in the other specified adenocarcinoma group and carcinosarcoma group, in whom prognosis is generally considered unfavorable. Considering the timing of changes in medical circumstances in Japan, these findings indicate that comprehensive medical development including the introduction of concomitant therapy with taxane and platinum might have contributed to these improvements.

## APPENDIX A. SUPPLEMENTARY DATA

eFigure 1. Relative survival of patients with endometrioid adenocarcinoma (A), other specified adenocarcinoma (B), and carcinosarcoma (C) in the 1st period (1993–2000) and the 2nd period (2001–2006). Dashed line, relative survival curve in the 1st period (1993–2000); solid line, relative survival curve in the 2nd period (2001–2006). (A) One-year RS of 1st and 2nd period were 94.0% and 97.0%. Five-year RS of 1st and 2nd period were 84.5% and 89.7%. (B) One-year RS of 1st and 2nd period were 86.5% and 90.4%. Five-year RS of 1st and 2nd period were 69.6% and 67.5%. (C) One-year RS of 1st and 2nd period were 65.9% and 72.1%. Five-year RS of 1st and 2nd period were 41.7% and 40.9%. OSA, other specified adenocarcinoma; RS, relative survival.
