# Supplementary material for: Improvement in 5-Year Relative Survival in Cancer of the Corpus Uteri From 1993–2000 to 2001–2006 in Japan
Source: J Epidemiol. 2018 Feb 5;28(2):75–80. doi: 10.2188/jea.JE20170008 (PMC5792230; doi:10.2188/jea.JE20170008)
Supplement: Supplementary file 1 [file je-28-075-s001.pdf]

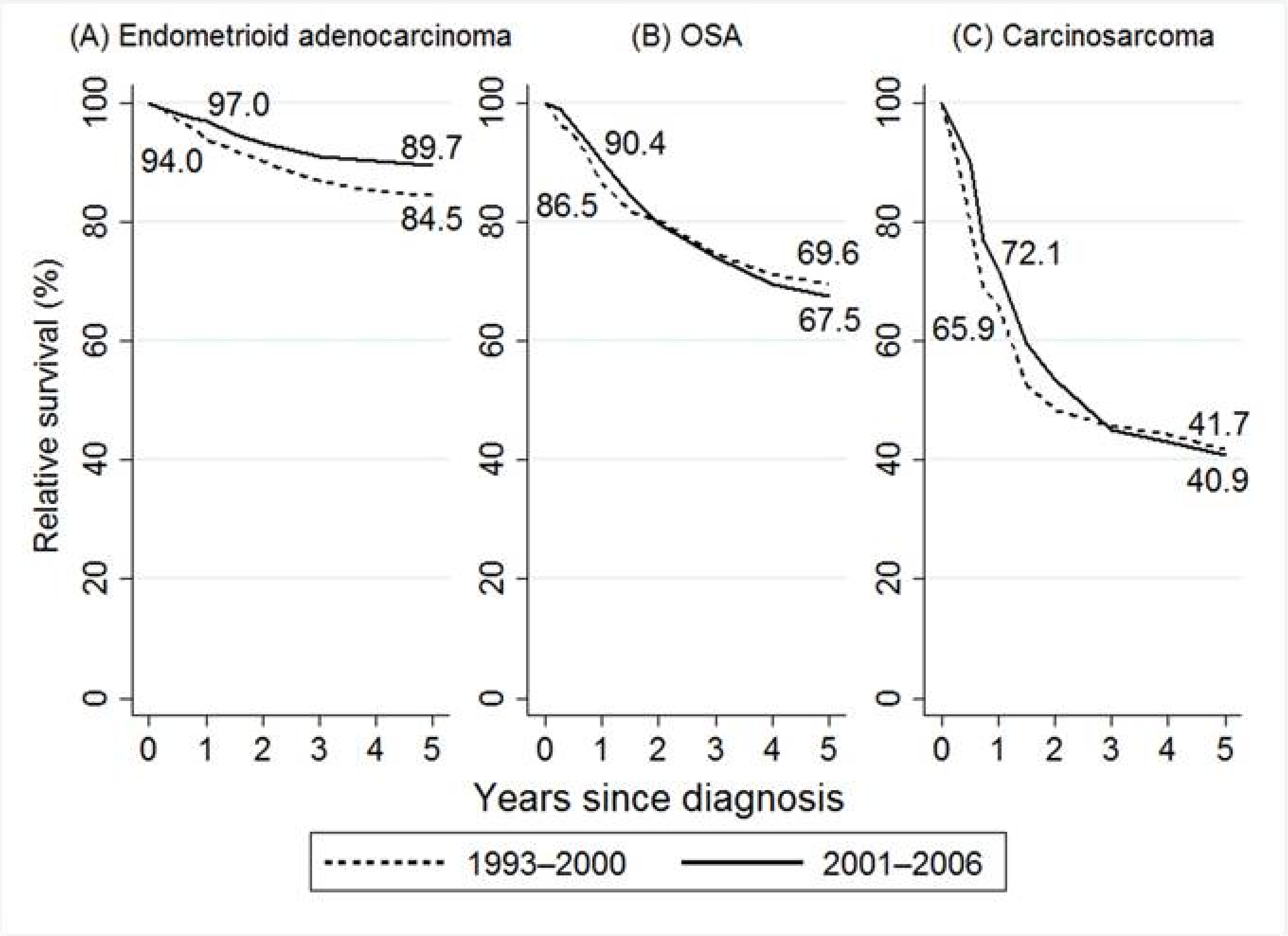

eFigure 1. Relative survival of patients with endometrioid adenocarcinoma (A), other specified adenocarcinoma (B), and carcinosarcoma (C) in the 1st period (1993–2000) and the 2nd period (2001–2006). Dashed line, relative survival curve in the 1st period (1993–2000); solid line, relative survival curve in the 2nd period (2001–2006). (A) One-year RS of 1st and 2nd period were 94.0% and 97.0%. Five-year RS of 1st and 2nd period were 84.5% and 89.7%. (B) One-year RS of 1st and 2nd period were 86.5% and 90.4%. Five-year RS of 1st and 2nd period were 69.6% and 67.5%. (C) One-year RS of 1st and 2nd period were 65.9% and 72.1%. Five-year RS of 1st and 2nd period were 41.7% and 40.9%. OSA, other specified adenocarcinoma; RS, relative survival.
